# Phenolic and Volatile Composition and Antioxidant Properties of the Leaf Extract of *Brassica fruticulosa* subsp. *fruticulosa* (*Brassicaceae*) Growing Wild in Sicily (Italy)

**DOI:** 10.3390/molecules27092768

**Published:** 2022-04-26

**Authors:** Emilia Cavò, Maria Fernanda Taviano, Federica Davì, Francesco Cacciola, Yassine Oulad El Majdoub, Luigi Mondello, Monica Ragusa, Concetta Condurso, Maria Merlino, Antonella Verzera, Natalizia Miceli

**Affiliations:** 1 Department of Chemical, Biological, Pharmaceutical and Environmental Sciences, University of Messina, Viale Ferdinando Stagno d’Alcontres 31, 98166 Messina, Italy; ecavo@unime.it (E.C.); federicadavi95@gmail.com (F.D.); yassine.ouladelmajdoub@unime.it (Y.O.E.M.); lmondello@unime.it (L.M.); nmiceli@unime.it (N.M.); 2 Foundation “Prof. Antonio Imbesi”, University of Messina, Piazza Pugliatti 1, 98122 Messina, Italy; 3 Department of Biomedical, Dental, Morphological and Functional Imaging Sciences, University of Messina, Via Consolare Valeria, 98125 Messina, Italy; cacciolaf@unime.it; 4 Chromaleont s.r.l., c/o Department of Chemical, Biological, Pharmaceutical and Environmental Sciences, University of Messina, Viale Palatucci, 98168 Messina, Italy; 5 Department of Sciences and Technologies for Human and Environment, University Campus Bio-Medico of Rome, Via Àlvaro del Portillo 21, 00128 Rome, Italy; 6 Complex Structure of Surgical Sciences and Technologies, IRCCS Istituto Ortopedico Rizzoli, Via di Barbiano 1/10, 40136 Bologna, Italy; monica.ragusa@ior.it; 7 Department of Veterinary Sciences, University of Messina, Viale Palatucci, 98168 Messina, Italy; concetta.condurso@unime.it (C.C.); maria.merlino@unime.it (M.M.); antonella.verzera@unime.it (A.V.)

**Keywords:** *Brassica fruticulosa* subsp. *fruticulosa*, edible plant, phenolic compounds, volatile compounds, antioxidant activity, *Artemia salina* Leach

## Abstract

In continuation of research conducted on species of the spontaneous flora of Sicily (Italy) belonging to the Brassicaceae family, *Brassica fruticulosa* subsp. *fruticulosa* was selected. It is an edible species utilized in Sicilian traditional medicine. In this study, for the first time, the phenolic and the volatile compounds and the antioxidant properties of the hydroalcoholic extract obtained from the leaves of *B. fruticulosa* subsp. *fruticulosa* were characterized. Through HPLC-PDA/ESI-MS analysis, a total of 22 polyphenolic compounds (20 flavonoids and 2 phenolic acids) were identified, with 3-hydroxiferuloylsophoroside-7-*O*-glucoside (1.30 mg/g ± 0.01) and kaempferol-3-*O*-feruloylsophoroside-7-*O*-glucoside (1.28 mg/g ± 0.01) as the most abundant compounds. Through SPME-GC/MS several volatiles belonging to different chemical classes were characterized, with nitriles and aldehydes accounting for more than 54% of the whole volatile fraction. The extract of *B. fruticulosa* subsp. *fruticulosa* showed moderate activity in the DPPH assay (IC_50_ = 1.65 ± 0.08 mg/mL), weak reducing power (17.47 ± 0.65 ASE/mL), and good chelating properties (IC_50_ = 0.38 ± 0.02 mg/mL), reaching approximately 90% activity at the highest tested concentration. Lastly, the extract was non-toxic against *Artemia salina*, indicating its potential safety. According to the findings, it can be stated that *B. fruticulosa* subsp. *fruticulosa* represents a new valuable source of bioactive compounds.

## 1. Introduction

The Brassicaceae family (also called Cruciferae), order Brassicales, consisting of more than 300 genera and about 3500 species, includes a large number of vegetable crops recognized as rich sources of health-promoting phytochemicals [1,2].

*Brassica* is the economically most important genus within the tribe Brassiceae. Most of the *Brassica* species are cultivated throughout the world due to their economic, nutritional, medicinal, and pharmaceutical value. Nevertheless, the current increasing demand of medicinal plants for pharmaceuticals, nutraceuticals, cosmetics, and other products, represents an opportunity for the valorization of wild species of *Brassica* so far little or no investigated.

Some wild *Brassica* species have been used for centuries as important sources of food as part of the Mediterranean diet, and various studies have documented the nutritional and medicinal properties of the edible wild plants with respect to the cultivated crops [3].

Wild *Brassica* species have great potential as sources of bioactive compounds; indeed, the adaptation to challenging environmental conditions has led the plants to direct greater resources to the synthesis of specialized secondary metabolites as a chemical defense mechanism [4].

In the last few years, the species belonging to the Brassicaceae family that grow spontaneously in Sicily have been investigated by our research team to unearth new valuable plant sources of bioactive compounds. Recently, our team reported the characterization of the phenolic components, as well as the antioxidant and cytotoxic properties, of the leaf and flowering top extracts of *Brassica incana* Ten. [5]. In continuation of our studies, we selected *Brassica fruticulosa* Cyr. subsp *fruticulosa*, a species not fully studied so far.

*Brassica fruticulosa* subsp *fruticulosa* (Mediterranean cabbage) is a species with Mediterranean distribution, but with smaller representation in Europe, Southwest Asia, Central and Southern Africa, and the eastern coast of North America [6,7]. This species is widespread in southern Italy, and it grows in untilled lands, as well as on walls and debris, from 0 to 1200 m above the sea level [8,9].

*Brassica fruticulosa* Cyr. subsp. *fruticulosa*, included in the subgen. *Brassica*, sect. *Micropodium* DC. [10], is an herbaceous species, usually biennial to perennial, 20–60 cm high; it presents a suffruticose aspect with a woody stem at the base. The basal leaves are long-petiolate, lirate, and arranged to form a rosette; the cauline leaves are smaller, pinnate-lobed to entire. It blooms from January to December, and it has flowers gathered in racemes with violet sepals and yellow petals. The fruit is a siliqua constricted at intervals, stipitate, with a beak of 2–7 mm [11,12].

This species is widely diffused in Sicily (Italy), where its use in traditional medicine is reported; indeed, the leaf decoction of *B. fruticulosa* subsp. *fruticulosa* is utilized to raise blood pressure and as an antidiabetic [13,14].

Furthermore, *B. fruticulosa* subsp. *fruticulosa* is an edible plant; this species has been eaten since ancient times both raw and cooked. The edible portion is represented by young shoots and leaves which are picked up until they are tender, before flowering, and commercialized in local markets during October–April. In southern Italy, especially in Sicily, cooked leaves and young shoots of *B. fruticulosa* subsp. *fruticulosa* are utilized to prepare traditional dishes [8]. Typical dishes include shoots boiled and dressed with olive oil and lemon juice or stir-fried with garlic and chili pepper, as a side dish to pork sausages [15,16]. Its use for the preparation of a typical Sicilian polenta, known as “Frascatula”, together with *Brassica incana* and other wild herbs, is reported in Sicily [5].

Concerning phytochemical composition, some studies have been carried out on the leaves, roots, and seeds of this species [8,17,18,19,20]. To the best of our knowledge, no investigations about the biological properties of *B. fruticulosa* subsp. *fruticulosa* are reported.

The present work was undertaken to characterize the phenolic and volatile constituents, and to investigate the antioxidant properties and potential toxicity of a hydroalcoholic extract obtained from the leaves of *B. fruticulosa* subsp. *fruticulosa* grown wild in Sicily (Italy). In particular, the qualitative–quantitative profile of the phenolic and volatile constituents contained in the extract was obtained by HPLC-PDA/ESI-MS and SPME-GC/MS analyses. The antioxidant properties were examined by means of different in vitro systems: DPPH (2,2-diphenyl-1-picrylhydrazyl) scavenging, reducing power, and ferrous ion (Fe^2+^)-chelating activity. Lastly, the toxicity of the extract was assessed by the brine shrimp (*Artemia salina* Leach) lethality bioassay.

## 2. Results and Discussion

### 2.1. Phytochemical Investigations

#### 2.1.1. Determination of Total Phenolic Content

The Folin–Ciocâlteu assay is a recognized, widely used procedure for quantification of total phenolic compounds in plant extracts. It is a colorimetric method based on electron transfer reactions between the Folin–Ciocâlteu reagent and phenolics, giving rise to the formation of a blue chromophore with the maximum absorption at 765 nm. Generally, gallic acid is used as the reference standard compound, and the results are usually expressed as gallic acid equivalent [21]. In most cases, the antioxidant properties of plant extracts are explained by their total phenolic content with good correlation, confirming the value of this assay. Therefore, the determination of their total amount in the extract used for this study was performed.

The results of the Folin–Ciocâlteu assay showed that the total phenolic content of *B. fruticulosa* subsp. *fruticulosa* leaf extract was equal to 32.63 ± 1.11 mg gallic acid equivalent (GAE)/g extract. This content was close to that of the *B. incana* leaf extract previously investigated (37.20 ± 0.93 mg GAE/g extract) [5].

#### 2.1.2. Identification of Phenolic Compounds by HPLC-PDA/ESI-MS

**Figure 1:**
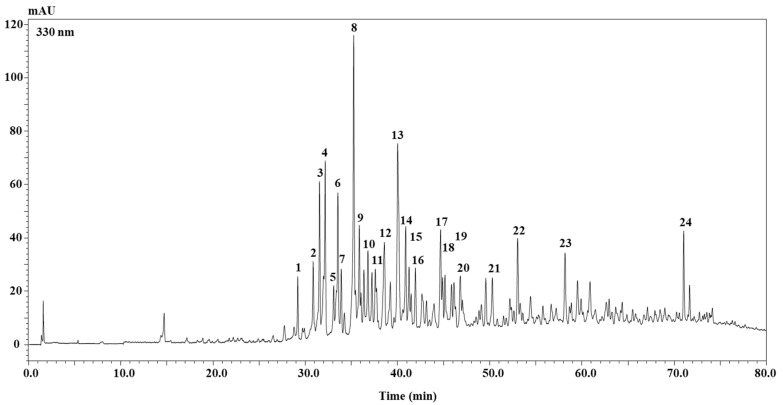
HPLC-PDA chromatograms of the polyphenolic compounds, extracted at 330 nm wavelength, of *B. fruticulosa* subsp. *fruticulosa* leaf hydroalcoholic extract. For peak identification, see Table 1.

For the first time, the phenolic profile of the hydroalcoholic extract of the leaf of *B. fruticulosa* subsp. *fruticulosa* is reported. The HPLC-PDA chromatogram (λ = 330 nm) of the polyphenolic compounds occurring in the extract is shown in Figure 1. A total of 24 compounds were detected and, among them, according to retention times, as well as PDA, MS and MS/MS, and literature data, 22 were tentatively identified (Table 1) [5,22,23,24,25,26,27,28]. Notably, most of them belonged to the flavonoid class, whereas only two were phenolic acids. Among flavonoids, 10 were kaempferol derivates, nine were quercetin derivates, and only two were isorhamnetin derivates. With regard to the two phenolic acids, they were sinapic and ferulic hydroxycinnamic acids in conjugation with a gentiobiose moiety.

As can be seen from Table 1, among the phenolic compounds identified, flavonols represented the most abundant constituents (11.1 mg/g extract), while phenolic acids were not quantified. Many of the compounds identified were previously reported to be constituents of *Brassica juncea* L. or *B. incana* [5,22].

Regarding quantification, since none of the compounds identified were commercially available, three selected reference standards were considered, namely, quercetin-3-*O*-glucoside, kaempferol-3-*O*-glucoside, and isorhamnetin-3-*O*-glucoside, for the determination of quercetin, kaempferol, and isorhamnetin derivates, respectively. Results were expressed as standard mg/g extract (dw) ± relative standard deviation (% RDS). Notably, peak no. 8, namely, kaempferol 3-hydroxyferuloylsophoroside-7-*O*-glucoside, turned out to be the most abundant (1.30 mg/g ± 0.01), followed by peak no. 13, kaempferol-3-*O*-feruloylsophoroside-7-*O*-glucoside (1.28 mg/g ± 0.01).

In a previous study, we characterized the polyphenol compounds contained in the leaves of another *Brassica* wild species from Sicily, namely, *B. incana*, utilizing the same procedure of extraction reported here. By comparing the polyphenol profile of the leaf hydroalcoholic extract of *B. fruticulosa* subsp. *fruticulosa* with that *of B. incana*, a similar flavonoid pattern could be appreciated between the two species, with derivatives of the flavonols quercetin, kaempferol, and isorhamnetin, together with the hydroxycinnamic acids sinapic acid and ferulic acid. Nonetheless, some differences among the two species were highlighted; indeed, a greater number of flavonoid derivatives were detected in the *B. fruticulosa* subsp *fruticulosa* leaf extract, whereas the hydroxycinnamic acids identified in the *B. incana* extract were found to be more numerous and in conjugation with malic acid and glucose moieties, in addition to gentiobiose [5].

#### 2.1.3. Identification of Volatile Compounds by SPME-GC/MS

The volatile composition of the hydroalcoholic extract of the aerial parts of *B. fruticolosa* subsp. *fruticulosa* is reported in Table 2. Many compounds, such as esters, alcohols, acids, ketones, aldehydes, terpenes, hydrocarbons, sulfur compounds, and nitriles, were determined. Nitriles (35.08%) and aldehydes (19.67%) constituted more than 54% of the whole volatile fraction; terpenoids (12.11%) and ketones (11.06%) were also quantitatively well represented. Among sulfur compounds, no isothiocyanates were detected, with only dimethyl disulfide and dimethyl trisulfide identified.

The main volatile compounds were 3-methyl-3-butenenitrile, heptanenitrile and benzenepropane nitrile, hexanal, 2-methyl-2-nonen-4-one, and 10-(acetylmethyl)-(+)-3-carene.

The identified compounds are well known secondary metabolites of plants [27] and, in particular, nitriles are common in *Brassica* species. Indeed, nitriles, as well as isothiocyanates, originate from the hydrolysis of glucosinolates, a group of compounds typical of Brassicaceae, Capparaceae, and Caricaceae families. The enzyme myrosinase, released upon tissue damage, hydrolyzes the β-d-*S*-glycosidic bonds of glucosinolates, releasing the sulfur-containing aglycone moieties that are unstable and undergo the Lossen rearrangement to form various breakdown products such as isothiocyanates, thiocyanates, nitriles, epithionitriles, and oxazolidine-2-thiones. The products of glucosinolate hydrolysis depend on various factors, such as the glucosinolate substrate, the reaction conditions, the presence of substances which can modify the action of the enzyme, and the plant pretreatments. It has been demonstrated that, if the hydrolytic reaction occurs under acidic conditions, low temperature, and low water levels, nitrile formation is favored. Nitriles are also favored by autolysis, rather than by the action of an exogenous source of the enzyme and by the presence of ferrous ion. Moreover, in fresh or freeze–thawed leaves, the glucosinolate hydrolysis produced mainly nitriles, whereas dry heating of the leaves decreased the proportion of nitrile formation and increased the proportion of isothiocyanate formation [29].

Although few data are present in the literature on the glucosinolate composition of *B. fruticolosa* [30], with none referring to leaves, the nitriles here identified are consistent with the structure of glucosinolates previously reported in Brassicaceae [31].

Among the minor constituents, safranal, β-cyclocitral, and β-ionone originate from the enzymatic breakdown of carotenoids. These terpenoids have been detected in the flowering top extract of *B. incana* [32] and in the hydroalcoholic extract of the aerial part of different *Matthiola* species, such as *M. fruticolosa* [28] and *M. tricuspidata* (our unpublished data). Furthermore, hexahydrofarnesyl acetone or phytone was detected in our previous studies on hydroalcoholic extract of *B. incana* and *Matthiola* spp. [28,32,33]; this ketone very common in plants arises from the oxidative degradation of (*E*)-phytol, a diterpene alcohol that occurs as a side-chain of chlorophyll a [34].

The results here reported are quite different from those described in our previous study on the volatiles of *B. fruticolosa* leaves [8]. This can be explained considering that we previously applied the SPME technique directly to the plant leaves, whereas, in this case, a hydroalcoholic extract of the leaves was considered.

Considering the volatile profile of the leaf hydroalcoholic extract of another *Brassica* species, namely, *B. incana*, only a few qualitative similarities emerged, whereas, from a quantitative point of view, in the volatile profile of *B. incana* leaf extract, isothiocyanates prevailed vs. nitriles [32].

### 2.2. Antioxidant Activity

Oxidative stress has been identified as the root cause of the development and progression of many diseases. In recent years, several epidemiological studies have indicated that a high intake of plant products is associated with a reduced risk of a number of chronic diseases, such as atherosclerosis and cancer, partly attributed to the compounds which possess antioxidant activity [35]. Bibliographic data show that many species belonging to the genus *Brassica* contain phenolic compounds, widely considered to be the most important specialized metabolites with antioxidant activity [24].

Antioxidant activity should be evaluated by the use of various methods in order to acquire a more complete antioxidant profile. In these assays, plant extracts are generally assessed for their function as reducing agents, hydrogen donors, singlet oxygen quenchers, or metal chelators [36]. When they react with free radicals by producing less reactive species or by interrupting the radical chain reaction, they are classified as primary (chain breaking) antioxidants; on the contrary, when they act by suppressing the formation of radicals and protecting against oxidative damage, they are defined as secondary (preventive) antioxidants [37]. Thus, three in vitro assays based on different approaches and mechanisms were used in order to determine the antioxidant capacity of *B. fruticulosa* subsp. *fruticulosa* extract. The primary antioxidant properties were examined using the DPPH assay, based on the hydrogen atom transfer (HAT) and electron transfer (ET) mechanisms, and the reducing power, an ET-based assay. The secondary antioxidant ability was determined by measuring the ferrous ion (Fe^2+^)-chelating activity.

**Figure 2:**
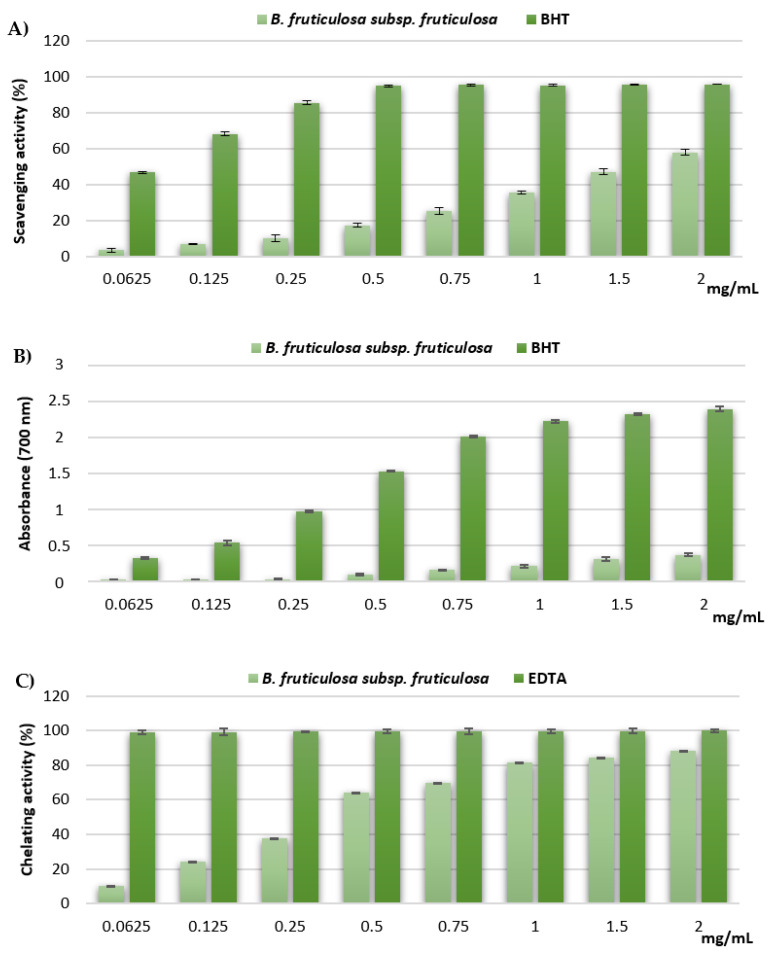
Free-radical-scavenging activity (DPPH assay) (**A**), reducing power (**B**), and ferrous ion-chelating activity (**C**) of *B. fruticulosa* subsp. *fruticulosa* leaf hydroalcoholic extract. Values are expressed as the mean ± SD (*n* = 3).

The results of the DPPH test, utilized to establish the free-radical-scavenging properties of the extract, are shown in Figure 2A. Compared with the reference standard BHT, the hydroalcoholic leaf extract of *B. fruticulosa* subsp. *fruticulosa* exhibited moderate scavenging activity, dose-dependently, in the range of concentrations assayed (0.0625–2 mg/mL), reaching about 60% inhibition of the DPPH radical at the highest concentration tested. The IC_50_ values confirmed the lower activity of the extract with respect to the standard BHT (1.65 ± 0.08 mg/mL and 0.07 ± 0.01 mg/mL, respectively).

From the comparison of the scavenging activity of the extract with that highlighted for *B. incana* leaf extract (IC_50_ = 1.31 ± 0.05 mg/mL), previously investigated under the same experimental conditions, it is evident that *B. fruticulosa* subsp. *fruticulosa* extract had a slightly lower activity [5].

The reducing power reflects the ability to stop the radical chain reaction. In this assay, the presence of antioxidant compounds in the sample determines the reduction of Fe^3+^ to the ferrous form (Fe^2+^); this reduction is highlighted by spectrophotometric measurement (700 nm) of the change of yellow color of the test solution to various shades of green and blue, depending on the reducing power of the antioxidant sample [38].

Figure 2B shows the results of the reducing power of the hydroalcoholic leaf extract of *B. fruticulosa* subsp. *fruticulosa*; the extract exhibited mild, concentration-dependent, reducing power, as compared with the standard BHT. This was confirmed also by the ASE/mL values (17.47 ± 0.65 and 0.89 ± 0.06 ASE/mL, respectively). This result agrees with that previously reported for the extract of *B. incana* leaves [5].

The method of Fe^2+^-chelating activity utilized the reagent ferrozine, which can quantitatively form complexes with Fe^2+^; in the presence of chelating agents, the complex formation is inhibited, with the result that the red color of the complex is decreased. Measurement of color reduction, therefore, allows the estimation of the chelating activity of the coexisting chelator [38].

*Brassica fruticulosa* subsp. *fruticulosa* extract exhibited strong and dose-dependent chelating properties (Figure 2C), reaching approximately 90% activity at the highest tested concentration. Nevertheless, the extract was not as effective as the reference standard EDTA (IC_50_ = 0.38 ± 0.02 and 0.007 ± 0.001 mg/mL, respectively). In comparison with *B. incana* leaf extract, previously investigated under the same experimental conditions (IC_50_ = 1.147 ± 0.016 mg/mL), *B. fruticulosa* subsp. *fruticulosa* extract exhibited much higher chelating properties [5].

The results of the in vitro antioxidant tests showed that *B. fruticulosa* subsp. *fruticulosa* extract acts as moderate primary antioxidant and possesses strong secondary antioxidant properties.

Taking into consideration that flavonoids are known to display metal-chelating effects [39], the good chelating activity of the extract may depend to some extent on the presence of flavonol derivatives, mostly of quercetin and kaempferol, detected by HPLC-PDA/ESI-MS analysis; however, the involvement of other polar constituents present in the phytocomplex cannot be excluded.

### 2.3. Artemia salina Leach Lethality Bioassay

The toxicity of the extract was assessed by the *Artemia salina* Leach bioassay. The brine shrimp lethality bioassay is extensively utilized as an alternative model for toxicity evaluation because it offers numerous advantages such as rapidity, cost-effectiveness, continuous availability of cysts (eggs), and ease of handling and maintenance under laboratory conditions [40]. It represents a simple technique for predicting the toxicity of plant extracts in order to consider their safety. The results of the bioassay showed the absence of toxicity against brine shrimp larvae for the extract of *B. fruticulosa* subsp. *fruticulosa*. Indeed, the median lethal concentration values were found to be above 1000 µg/mL, thus indicating their potential safety according to Clarkson’s toxicity criterion [41]. These data are in agreement with those observed for the extracts of *B. incana* investigated in our previous work [5].

## 3. Materials and Methods

### 3.1. Chemicals and Reagents

LC–MS-grade water (H_2_O), acetonitrile (ACN), isorhamnetin-3-*O*-glucoside, quercetin-3-*O*-glucoside, and kaempferol-3-*O*-glucoside were obtained from Merck Life Science (Merck KGaA, Darmstadt, Germany). LC–MS-grade formic acid was purchased from Riedel-de Haën (Seelze, Germany). Methanol (MeOH) was purchased from Carlo Erba (Milan, Italy). Unless indicated otherwise, all chemicals were purchased from Sigma-Aldrich (Milan, Italy).

### 3.2. Plant Material and Extraction Procedure

The leaves of the *Brassica fruticulosa* subsp. *fruticulosa* were collected in the locality of Massa San Giorgio, on the Peloritani Mountains (Messina, Sicily, Italy), in October 2019. The taxonomic identification was confirmed by Prof. S. Ragusa, Department of Health Sciences, University Magna Graecia of Catanzaro (Catanzaro, Italy). A voucher specimen (1016/19) was deposited in the same Department.

After harvesting, the plant material was washed, blended, frozen, and then lyophilized. Subsequently, the leaves, finely ground, were subjected to a preventive maceration at 25 °C with 70% MeOH (1:10 *w*/*v*) for 1 h. The extraction was performed with 70% MeOH (1:10 *w*/*v*) in an ultrasonic bath at 50 °C for 15 min, repeated three times; then, the filtrates were combined and evaporated to dryness by a rotavapor. The yield of the leaf extract, referring to 100 g of lyophilized plant material, was 22.99%.

### 3.3. Phytochemical Investigation

#### 3.3.1. Determination of Total Phenolic Content

The total phenolic content of *B. fruticulosa* subsp. *fruticulosa* leaf extract was determined by the Folin–Ciocâlteu colorimetric method, using gallic acid as a standard phenolic compound [42]. An aliquot of 0.1 mL of each sample solution was mixed with 0.2 mL Folin–Ciocâlteu reagent, 2 mL of distilled water, and 1 mL of 15% Na_2_CO_3_. A linear calibration curve of gallic acid, in the range 125–500 µg/mL, was constructed. The absorbance was measured at 765 nm, after a 2 h incubation at room temperature, with a UV-1601 spectrophotometer (Shimadzu, Milan, Italy). The total phenolics were expressed as mg GAE/g of extract (dw) ± standard deviation (SD). The data were obtained from the average of three independent determinations.

#### 3.3.2. Identification of Phenolic Compounds by HPLC-PDA/ESI-MS

The analyses were carried out using a Shimadzu HPLC system (Milan, Italy) equipped with a CBM-20A controller, LC-20AD pumps, a DGU-20A3 degasser, a SIL-20AC autosampler, an SPD-M20A photo diode array detector (PDA), and a triple-quadrupole mass analyzer (LCMS-8050, Shimadzu, Kyoto, Japan), equipped with an ESI interface, in positive and negative ionization mode. Data acquisition was performed by Shimadzu LabSolution software ver. 5.91.

##### Samples and Sample Preparation

*B. fruticulosa* subsp. *fruticulosa* leaf extract (30.5 mg) was dissolved in 100 µL of MeOH.

##### Chromatographic Conditions

Analyses were carried out on a Ascentis Express C18, 15 cm × 4.6 mm internal diameter (i.d.), with particle size of 2.7 µm (Merck Life Science, Merck KGaA, Darmstadt, Germany). The injection volume was 5 µL, and the mobile phase consisted of water/formic acid (99.9:0.1, *v*/*v*) (solvent A) and ACN/formic acid (99.9:0.1, *v*/*v*) (solvent B); the linear gradient profile was as follows: 0 min, 0% B; 15 min, 5% B; 65 min, 20% B; 95 min, 35% B; 100 min, 100% B; 101 min, 0% B. The flow rate for separation and detection was 1 mL/min, and it was split to 0.2 mL/min prior to MS detection.

##### PDA Conditions

The wavelength range was 200–400 nm, and the chromatograms were extracted at 280 nm. The time constant was 0.08 s, and the sample frequency was 40 Hz.

##### MS Conditions

The MS acquisition was performed using the ESI interface in negative ionization mode. Mass detection was performed in full scan mode in the spectral range 100–1400 *m*/*z*, with an interval of 0.5 s. Nitrogen (N_2_) was used as a nebulizing gas at a flow rate of 3 L/min. The following settings were applied to the instrument: interface temperature, 300 °C; heat block, 400 °C; DL temperature, 250 °C; DL voltage, −34 V; probe voltage, 4.5 kV; Q-array voltage, 1.0 V; RF voltage, 90 V; detection gain, 1.0 kV.

Quantitative determination was carried using calibration curves of three standards, representative of the chemical classes under study, namely, isorhamnetin-3-*O*-glucoside (*y* = 14948*x* − 2966.9; limit of detection (LOD) = 0.032, limit of quantification (LOQ) = 0.098), quercetin-3-*O*-glucoside (*y* = 13424*x* + 898.59; LOD = 0.013, LOQ = 0.043), and kaempferol-3-*O*-glucoside (*y* = 17660*x* − 10681; LOD = 0.023, LOQ = 0.072). Standard calibration curves were prepared in a concentration range 0.1–1000 mg/L with five different concentration levels.

#### 3.3.3. Identification of Volatile Compounds by SPME-GC/MS

##### Extraction (HS-SPME)

The leaf extract of *B. fruticolosa* subsp. *fruticulosa* was analyzed for its volatile composition by HS-SPME-GC/MS as previously reported [28,33,43].

The dried extract was solubilized in saturated sodium chloride solution to a final concentration of 10 mg/mL; then, 3 ± 0.1 mL of each extract solution was transferred to a 7 mL vial closed with a ‘mininert’ valve (Supelco, Bellefonte, PA, USA). For the volatile extraction, the sample was equilibrated for 15 min at 40 °C, and a DVB/CAR/PDMS fiber, 50/30 μm film thickness (Supelco, Bellefonte, PA, USA), was exposed for 15 min to the headspace of the sample maintained at 40 °C under continuous magnetic stirring. Finally, the SPME fiber was placed for 3 min into the injector port of the GC/MS, held at 260 °C, for the thermal desorption of the analytes onto the capillary GC column.

##### Analysis (GC/MS)

The volatiles were analyzed by a Shimadzu GC 2010 Plus gas chromatograph coupled to a TQMS 8040 triple-quadrupole mass spectrometer (Shimadzu, Milan, Italy). Two capillary columns of different polarity were used: (1) a VF-WAXms, 60 m, 0.25 mm i.d., 0.25 μm film thickness polar column (Agilent Technologies Italia S.p.A., Milan, Italy); (2) a DB-5 ms, 30 m, 0.25 mm i.d., 0.25 μm film thickness apolar column (Agilent Technologies Italia S.p.A., Milan, Italy).

The conditions were as follows: injection mode, splitless; oven temperature (1) 45 °C held for 5 min, then increased to 80 °C at a rate of 10 °C/min and to 240 °C at 2 °C/min, held at 240 °C for 5 min for polar column, (2) 45 °C increased to 160 °C at a rate of 3 °C/min and to 260 °C at 10 °C/min, held at 260 °C for 5 min for apolar column; carrier gas, helium at a constant flow of 1 mL/min; transfer line temperature, 250 °C; acquisition range, 40 to 360 m/z; scan speed, 1250. For the identification of the volatiles, mass spectral data, NIST’ 14 (NIST/EPA/NIH Mass Spectra Library, version 2.0, Gaithersburg, MD, USA) and FFNSC 3.0 database, and linear retention indices (LRI) were used.

### 3.4. Antioxidant Activity

#### 3.4.1. Free-Radical-Scavenging Activity

The free-radical-scavenging activity of the hydroalcoholic leaf extract of *B. fruticulosa* subsp. *fruticulosa* was evaluated using the DPPH (2,2-diphenyl-1-picrylhydrazyl) test [44]. DPPH is a stable radical in methanol with violet color because of delocalization of the spare electron throughout the molecule. When a proton is accepted in the reaction with the oxygen atom of a radical scavenger’s OH group, the reduced DPPH-H (2,2-diphenyl-1-picrylhydrazine) is formed, which is yellow. The degree of discoloration indicates the amount of DPPH scavenged; a greater bleaching action indicates higher antioxidant activity, as reflected in a lower IC_50_ value.

The extract was tested at different concentrations (0.0625–2 mg/mL) using butylated hydroxytoluene (BHT) as a reference compound. A volume of 0.5 mL of each sample solution was mixed with 3 mL of daily prepared methanol DPPH solution (0.1 M) and incubated for 20 min at room temperature in the dark. Then absorbance was measured at 517 nm using a model UV-1601 spectrophotometer (Shimadzu, Milan, Italy). The scavenging activity was measured as the decrease in absorbance of the samples versus DPPH standard solution. Results were obtained from the average of three independent experiments, and they were expressed as the mean radical-scavenging activity percentage (%) ± SD and mean 50% inhibitory concentration (IC_50_) ± SD.

#### 3.4.2. Reducing Power Assay

The reducing power of the hydroalcoholic leaf extract of *B. fruticulosa* subsp. *fruticulosa* was determined according to the Fe^3+^–Fe^2+^ transformation method [45]. The extract was tested in the range of 0.0625–2 mg/mL. A volume of 1 mL of each sample was mixed with 2.5 mL of phosphate buffer (0.2 M, pH 6.6) and 2.5 mL of 1% potassium ferricyanide (K_3_Fe(CN)_6_). Following incubation at 50 °C for 20 min and rapid cooling, 2.5 mL of 10% trichloroacetic acid was added, and the mixture was centrifuged (3000 rpm, 10 min). Finally, 2.5 mL of the supernatant was mixed with 2.5 mL of distilled water and 0.5 mL of 0.1% ferric chloride (FeCl_3_). After incubation for 10 min of at room temperature in the dark, the color change of the sample was estimated by measuring absorbance at 700 nm. The increased absorbance of the reaction mixture indicates an increase in reducing power. Ascorbic acid and butylated hydroxytoluene (BHT) were used as reference compounds. The results were obtained from the average of three independent experiments, and they were expressed as the mean absorbance values (700 nm) ± SD and ascorbic acid equivalent/mL of extract (ASE/mL) ± SD.

#### 3.4.3. Ferrous Ion (Fe^2+^)-Chelating Activity Assay

The Fe^2+^-chelating activity of the hydroalcoholic leaf extract of *B. fruticulosa* subsp. *fruticulosa* was estimated by measuring the formation of the Fe^2+^–ferrozine complex [46]. The extract was tested in the range of 0.0625–2 mg/mL, and ethylenediaminetetraacetic acid (EDTA) was used as positive control. A volume of 1 mL of each sample was mixed with 0.5 mL of MeOH and 50 µL of 2 mM FeCl_2_. Then, 0.1 mL of 5 mM ferrozine was added to initiate the reaction; the mixture was shaken vigorously and incubated at room temperature in the dark for 10 min. The control contained FeCl_2_ and ferrozine, which are complex formation molecules. The color change of the solutions was estimated by measuring absorbance spectrophotometrically at 562 nm. The results were obtained from the average of three independent experiments, and they were expressed as the mean inhibition of the ferrozine–Fe^2+^ complex formation (%) ± SD and IC_50_ ± SD.

### 3.5. Artemia Salina Leach Lethality Bioassay

The *Artemia salina* Leach (brine shrimp) lethality bioassay was employed to predict the toxicity of the leaf hydroalcoholic extract of *B. fruticulosa* subsp. *fruticulosa* [47]. Brine shrimp eggs were hatched in artificial seawater (33 g sea salt/L deionized water) by incubation under a 60 W lamp, providing direct light and warmth (24–26 °C). After hatching, 10 brine shrimp larvae were incubated at 25–28 °C in 5 mL of artificial seawater mixed with different amounts of the extract (10, 100, 500, and 1000 µg/mL). After 24 h, the numbers of surviving nauplii were counted using a magnifying glass. The experiments were conducted in triplicate for each concentration, and the median lethal concentration (LC_50_) values were determined by Litchfield and Wilcoxon’s method. The toxicity level of the extract was assessed according to the toxicity scale reported by Clarkson et al. [41]. Extractis considered non-toxic if the LC_50_ is higher than 1000 µg/mL.

## 4. Conclusions

This work described the results of the phytochemical characterization and the antioxidant properties of the leaf hydroalcoholic extract of *B. fruticulosa* subsp. *fruticulosa* growing wild in Sicily (Italy), never investigated before. An in-depth overview of the qualitative–quantitative composition of the phenolic and volatile constituents of the leaves was attained. On the basis of the in vitro antioxidant assays performed, it can be stated that the *B. fruticulosa* subsp. *fruticulosa* leaf extract had much higher secondary than primary antioxidant properties. Furthermore, the extract was found to be non-toxic against brine shrimp larvae, indicative of its potential safety.

The obtained results provide a substantial contribution to the knowledge of *B. fruticulosa* subsp. *fruticulosa* so far little studied, indicating this wild edible species as a new valuable source of antioxidant compounds with potential health-promoting effects.

## Figures and Tables

**Table 1:** HPLC-PDA/ESI-MS (negative ionization mode) polyphenolic fingerprint of *B. fruticulosa* subsp. *fruticulosa* leaf hydroalcoholic extract. Results are expressed as mg/g extract ± SD (*n* = 3).

No.	t_R_(min)	UV _max_ (nm)	[M-H]^−^	Compound	mg/g ± %RSD	Ref.
1	29.19	254, 352	787, 625	Quercetin-3-*O*-sophoroside-7-*O*-glucoside	0.36 ± 0.001	[5,21]
2	30.87	340	979, 625	Quercetin-3-*O*- hydroxyferuoyl-sophoroside-7-*O*-D-glucoside	0.51 ± 0.002	[5,21]
3	31.57	264, 344	773, 609	Kaempferol-3-*O*-diglucoside-7-*O*-glucoside	0.75 ± 0.011	[5,21]
4	32.16	338	949, 301	Quercetin-3-caffeoylsophoroside-7-glucoside	0.86 ± 0.016	[5]
5	33.08	330	1111, 787	Quercetin-3-triglucoside-7-diglucoside	0.31 ± 0.001	[22]
6	33.53	328	963, 801	Kaempferol-3-*O*-hydroxyferuloylsophoroside-7-*O*-glucoside	0.53 ± 0.001	[5,21]
7	33.91	345	1125, 801	Kaempferol-3-*O*-hydroxyferuloyl diglucoside-7-*O*-diglucoside	0.32 ± 0.002	[23]
8	35.23	267, 331	933	Kaempferol-3-hydroxyferuloylsophoroside-7-*O*-glucoside	1.30 ± 0.003	[21]
9	35.83	334	1155, 831	Quercetin-3-sinapoyltriglucoside-7-glucoside	0.54 ± 0.012	[5,21]
10	36.76	338	963, 801	Quercetin-3-*O*feruloyldiglucoside-7-*O*-glucoside	0.63 ± 0.006	[23]
11	37.20	334	963, 801	Quercetin-3-O-feruloyldiglucoside-7-*O*-glucoside isomer	0.45 ± 0.011	[23]
12	38.53	268, 331	977, 815	Kaempferol-3-*O*-sinapoyldiglucoside-7-*O*-glucoside	0.65 ± 0.015	[23]
13	39.95	268, 331	947, 609	Kaempferol-3-*O*-feruloylsophoroside-7-*O*-glucoside	1.28 ± 0.011	[21]
14	40.82	267, 330	1019	Unknown	-	-
15	41.18	268, 318	917	Kaempferol-3-*O*-coumaroyl-sophoroside-7-*O*-d-glucoside	0.53 ± 0.002	[24]
16	41.88	349	639, 417, 315	Isorhamnetin-3-glucoside-7-glucoside	0.36 ± 0.005	[5,21]
17	44.61	326	753	Disinapoylgentiobiose	Nq	[5,21]
18	45.07	263, 343	625, 301	Quercetin-dihexoside	0.53 ± 0.021	[21,25]
19	46.06	324	723, 529	Sinapoylferuloylgentiobiose	Nq	[21,25]
20	46.70	335	787, 301	Quercetin-3-caffeoyisophoroside-7-glucoside	0.50 ± 0.001	[25]
21	50.17	264, 343	609, 285	Kaempferol-3-glucoside-7-glucoside	0.10 ± 0.001	[25]
22	52.91	266, 331	771, 285	Kaempferol-3-triglucoside	0.11 ± 0.001	[26]
23	58.00	268, 334	785, 285	Kaempferol-feruloyldihexoside	0.48 ± 0.004	[27]
24	70.92	327	1121	Unknown	-	-

Nq: Not quantified.

**Table 2:** Composition as volatile constituents and classes of substances of *B. fruticolosa* subsp. *fruticulosa* leaf hydroalcoholic extract.

Compounds	LRI * on DB-5ms	LRI * on VF-WAXms	Amount **	Percentage
**Sulfur compounds**				
Dimethyl disulfide	735	1078	460.369	2.77
Dimethyl trisulfide	957	1380	656.201	3.95
***All***			**1116.569**	***6.72***
**Nitriles**				
3-Methyl-3-butenenitrile	747	-	3487.559	20.98
5-Methylhexanenitrile	934	1349	438.854	2.64
Heptanenitrile	968	1406	924.253	5.56
Benzenepropane nitrile	1226	2041	980.772	5.90
***All***			**5831.437**	***35.08***
**Aldehydes**				
3-Methylbutanal	656	911	325.842	1.96
2-Methylbutanal	662	897	552.183	3.32
Hexanal	790	1082	708.226	4.26
(*E*)-2-Heptenal	948	1327	450.252	2.71
Benzaldehyde	951	1530	214.505	1.29
Octanal	994	1284	128.366	0.77
(*E,E*)-2,4-Heptadienal	1005	1508	128.722	0.77
Phenylacealdehyde	1033	1645	535.453	3.22
Nonanal	1094	1390	147.754	0.89
Decanal	1195	1491	77.683	0.47
***All***			**3268.984**	***19.67***
**Ketones**				
2,2,6-trimethylcyclohexanone	1049	1296	249.598	1.50
2-Methyl-2-nonen-4-one	1202	-	1158874	6.97
Hexahydrofarnesyl acetone	1825	2121	430.662	2.59
***All***			**1839.133**	***11.06***
**Alcohols**				
2-Ethyl-1-hexanol	1020	1483	126.165	0.76
(*E*)-2-Octen-1-ol	1059	1611	167.066	1.01
***All***			***293.231***	***1.76***
**Acids**				
Octanoic acid	1161	2062	503.754	3.03
Nonanoic acid	1257	2165	93.800	0.56
Decanoic acid	1355	2266	622.338	3.74
***All***			***1219.892***	***7.34***
**Esters**				
Ethyl octanoate	1186	1439	78.014	0.47
Ethyl decanoate	1382	1639	129.706	0.78
Ethyl dodecanoate	1580	1840	109.245	0.66
Methy tridecanoate	1612	1910	79.540	0.48
Ethyl tetradecanoate	1778	2040	26.940	0.16
Methyl hexadecanote	1905	2216	209.279	1.26
***All***			***632.724***	***3.81***
**Terpenoids**				
Safranal	1189	1649	520.036	3.13
β-Cyclocitral	1209	1626	400.886	2.41
10-(Acetyl methyl)-(+)-3-carene	1374	-	1030.412	6.20
(*E*)-β Ionone	1467	1928	62.218	0.37
***All***			***2013.551***	***12.11***
**Hydrocarbons**				
4,8-Dimethyl-1,7-nonadiene	1041	-	205.520	1.24
1,1,5-Trimethyl-1,2-dihydronaphthalene	1341	-	201.675	1.21
***All***			***407.194***	***2.45***

* Linear retention indices calculated according to the van den Dool and Kratz equation. ** Peak area arbitrary scale.

## Data Availability

The data presented in this study are available on request from the corresponding author.

## Sample Availability

Samples of the compounds are not available from the authors.
